# *Listeria monocytogenes*, a silent foodborne pathogen in Ecuador

**DOI:** 10.3389/fmicb.2023.1278860

**Published:** 2023-12-21

**Authors:** Lorena Mejía, Estefanía Espinosa-Mata, Ana Lucía Freire, Sonia Zapata, Fernando González-Candelas

**Affiliations:** ^1^Colegio de Ciencias Biológicas y Ambientales, Instituto de Microbiología, Universidad San Francisco de Quito USFQ, Quito, Ecuador; ^2^Institute for Integrative Systems Biology, University of Valencia, Valencia, Spain; ^3^Joint Research Unit “Infection and Public Health” FISABIO-University of Valencia, Valencia, Spain; ^4^CIBER (Centro de Investigación Biomédica en Red) in Epidemiology and Public Health, Valencia, Spain

**Keywords:** *Listeria monocytogenes*, foodborne, Ecuador, genomic epidemiology, whole genome sequencing

## Abstract

*Listeria monocytogenes* is a foodborne pathogen that can produce serious, even fatal, infections. Among other foods, it can be found in unpasteurized dairy and ready-to-eat products. Surveillance of *L. monocytogenes* is of great interest since sources of infection are difficult to determine due to the long incubation period, and because the symptoms of listeriosis are similar to other diseases. We performed a genomic study of *L. monocytogenes* isolated from fresh cheeses and clinical samples from Ecuador. Sixty-five isolates were evaluated and sequenced, 14 isolates from cheese samples and 20 from clinical listeriosis cases from the National Institute of National Institute of Public Health Research, and 31 isolates from artisanal cheese samples from 8 provinces. All isolates exhibited heterogeneous patterns of the presence of pathogenicity islands. All isolates exhibited at least 4 genes from LIPI-1, but all references (26 *L. monocytogenes* closed genomes available in the NCBI database) showed the complete island, which encompasses 5 genes but is present in only two Ecuadorian isolates. Most isolates lacked gene *actA*. Genes from LIPI-2 were absent in all isolates. LIPI-3 and LIPI-4 were present in only a few references and isolates. With respect to the stress survival islets, our samples either presented SSI-1 or SSI-F2365, except for one isolate that presented SSI-F2365 and also one gene from SSI-1. None of the samples presented SSI-2. The predominant ST (sequence type) was ST2 (84.62% 55/65), and the only ST found in food (93.33% 42/45) and clinical samples (65% 13/20). Isolates were not grouped according to their sampling origin, date, or place in a phylogenetic tree obtained from the core alignment. The presence of ST2 in food and clinical samples, with high genomic similarity, suggests a foodborne infection risk linked to the consumption of fresh cheeses in Ecuador.

## 1 Introduction

*Listeria monocytogenes* is a Gram-positive, facultative intracellular foodborne pathogen that can cause severe illnesses and conditions such as septicemia, encephalitis and meningitis, abortion, stillbirths, and neonatal infections in humans (Orsi et al., 2011) as well as severe disease in other mammals and birds (Jeffers et al., 2001). Although infections of *L. monocytogenes* are rare and the incidence of listeriosis is generally low, the case-fatality rate is one of the highest among foodborne infections (Seeliger, 1988), principally affecting young children, immunocompromised individuals, pregnant women, and the elderly (Bergholz et al., 2018).

*L. monocytogenes* can be found in both raw and processed foods, but the most implicated vehicles are usually ready-to-eat (RTE) food products such as cheeses, meat, seafood, and fresh products (Jordan and McAuliffe, 2018). Among these food matrices, cheeses are very important vehicles for *L. monocytogenes* transmission mainly because they are usually stored at refrigeration temperature (Walker et al., 1990) for several days, allowing the growth of this psychrophilic microorganism (Melo et al., 2015), and they do not always receive additional heat treatment before consumption. In a study about food outbreaks due to contaminated cheese (pasteurized and unpasteurized milk) in the USA from 1998 to 2011, even though *L. monocytogenes* was not the most common pathogen, it was the cause of death in five out of six cases (Gould et al., 2014).

*L. monocytogenes* represent a noticeable health threat when present in food and food-processing plants. It can persist in food-processing environments for long periods, due in part to its ability to survive under a great diversity of stressing conditions (sanitizing, pH, water activity, and temperature), and the ability to form biofilms on surfaces (Ferreira et al., 2014; Jordan et al., 2018). This bacterium can colonize food-processing facilities when contaminated raw materials are introduced, and when cross-contamination can occur between processed food and raw materials (Bolocan et al., 2016).

Although all isolates of *L. monocytogenes* are considered equally pathogenic by food regulatory authorities, certain serotypes are more frequently associated with clinical cases, as most human listeriosis outbreaks and sporadic cases are related to serotype 4b, and some less frequently with serotypes 1/2b and 1/2a (Orsi et al., 2011; Bergholz et al., 2018). Not only does the presence of different serotypes in food imply their association with clinical cases, but it is also now recognized that there are phenotypic differences between lineages and serotypes: the presence of virulence factors, ability to survive stress conditions and/or produce biofilms, presence of stop codons in virulence genes, among other genetic traits (Orsi et al., 2011; Hurley et al., 2019).

Whole genome sequencing (WGS) is a powerful tool for outbreak investigations and epidemiological surveillance (Hurley et al., 2019; Zhang et al., 2020). WGS allows an unprecedented subtyping resolution through the analysis of the core and accessory components of the genome to assess the genomic diversity, resistance determinant factors, virulence genotypes, stress resistance, and pathogenic potential (Moura et al., 2017; Hurley et al., 2019).

Listeriosis is a notifiable disease in North America and in many European countries, but not in Ecuador. Considering the serious consequences of listeriosis, its widespread distribution, and the absence of data from Ecuador, we used WGS to genotype and characterize *L. monocytogenes* isolates recovered from cheeses and clinical samples to assess the potential risk of this pathogen to cause disease in Ecuador.

## 2 Materials and methods

### 2.1 Bacterial strains

Sixty-five *Listeria monocytogenes* isolates were evaluated in this study. From the National Institute of Public Health Research in Ecuador, we had access to all *L. monocytogenes* isolates from the culture collection between 2015 and 2018 (14 isolates from cheese samples and 20 clinical isolates from listeriosis cases). We also had access to 31 isolates from artisanal cheese samples from 8 provinces in Ecuador from a previous study of our group (Espinosa-Mata et al., 2021). From these isolates, the antimicrobial susceptibility testing was assessed by microdilution technique, using panels of lyophilized antibiotics for Gram-positive bacteria (Sensititre GPALL1F, Thermo Scientific^®^) and their serogroup was determined by PCR as described by Tao et al. (2017) as presented by Espinosa-Mata and collaborators.

### 2.2 Whole genome sequencing

Genomic DNA from all the isolates was purified using Wizard Genomic DNA Purification Kit (Promega Corporation, Madison, WI, United States) following manufacturer’s guidelines for Gram positive bacteria. Next, an Invitrogen Qubit 3.0 fluorometer (Thermo Fisher Scientific, Walthman, MA, United States) was used for DNA quantification. DNA samples were sequenced with the Illumina NextSeq platform using Nextera XT Library Preparation Kit, and 150 × 2 bp paired-ends sequences were obtained. Trimmomatic (Bolger et al., 2014) was used for reads trimming, and Fastqc (Wingett and Andrews, 2018) and Multiqc (Ewels et al., 2016) were employed for quality assessment. Default parameters were used for the following software unless otherwise specified.

### 2.3 Sequence type determination, antimicrobial resistance genes, virulence factors, and plasmid detection

The software ARIBA version 2.11.1 (Hunt et al., 2017) was used with public databases CARD (Alcock et al., 2019), MEGAres (Doster et al., 2020), and ResFinder (Bortolaia et al., 2020) to identify genes that may confer antimicrobial resistance (AMR). The databases VirFinder for virulence genes, PlasmidFinder for plasmid determinants, and the PubMLST from Institut Pasteur (Moura et al., 2017) for *Listeria monocytogenes* for ST determination were also used with the ARIBA software.

We also wanted to determine the presence of plasmids previously described in other *L. monocytogenes* strains. We performed a manual search in public databases and downloaded 29 plasmids from NCBI. In order to see how similar these plasmids were, Proteinortho5 (Lechner et al., 2011) was used to determine orthology relationships, where 80% for coverage and 80% for similarity on protein sequences were set. With these results, we manually eliminated plasmids with similar gene patterns. Then, we selected 13 plasmids for the mapping step. We mapped our raw data against the plasmids following the strategy described in *Salmonella* megaplasmids detection (Mejía et al., 2020). With the fasta sequences from each strain, we performed an alignment with each plasmid in order to evaluate their coverage. Finally, we classified the plasmid presence in ranges of coverage for each strain (20–49%, 50–79%, and 80–100%). Plasmid coverages lower than 20% were discarded as absent. After analyzing the complete length covered by seven plasmids (pMF6172, pl2015TE24968, PAUSMDU00000224_01, pPIR00540, pLM58, pMF4545, pPIR00541), we performed an additional comparison based on orthologs and on average nucleotide identity (Yoon et al., 2017). We also used progressiveMauve (Darling et al., 2010) in order to identify local collinear blocks (LCB) and inversions.

### 2.4 Phylogenetic analysis

Spades (Nurk et al., 2013) were used to obtain the sequence assemblies from quality-trimmed reads. Twenty-six *L. monocytogenes* closed genomes were downloaded as references (Supplementary Table S1). Then, Prokka (Seemann, 2014) was used for genome annotation. Next, determinations of orthologs among all *L. monocytogenes* isolates and among ST2 isolates were performed independently with Proteinortho5 (Lechner et al., 2011) (using values of 90% for coverage and 80% for similarity on protein sequences). Genes in strict core for all isolates were extracted with the grab_proteins.pl. script from Proteinortho5. Then, all the genes were aligned and concatenated in a multifasta file with Mafft (Katoh, 2002) and an in-house script. The core genome alignment was used to obtain a maximum-likelihood phylogenetic tree with IQTree2 (Minh et al., 2019) with 1,000 ultrafast bootstrap replicates to evaluate branch supports (Hoang et al., 2018). iTOL (Letunic and Bork, 2019) was used to visualize the final trees and to add the metadata.

### 2.5 Genetic factors determination

In order to detect genes encoding major *Listeria* virulence factors (LIPI-1, LIPI-2, LIPI-3, LIPI-4, and internalins *inlA* and *inlB*), genes associated with biofilm formation (*flaA, luxS, cheY*), and genes from the stress survival islets (SSI-1, SSI-2, SSI-F2365), blastn was run with the reference genes (Supplementary Table S2) as subjects and the sequence assemblies as query. The coverage percentage for identification was set at 75%.

## 3 Results

### 3.1 Raw data

Raw sequence data is available under bioproject PRJEB48671. We obtained whole genome sequence data from 65 *L. monocytogenes,* 1 *L. seeligeri,* 1 *L. welshimeri,* and 5 *L. innocua* isolates. In this article, we focus mainly on *L. monocytogenes* isolates. The obtained average number of reads in millions per strain was 1.5 (range 0.3–2.6) (Supplementary Table S3), with an average Phred score of Q34. One *L. monocytogenes* isolate (Lm73) was excluded from subsequent analyses because the core genome of all isolates including Lm73 was composed of 1,227 orthologous genes, and without Lm73 it was composed of 2,112 genes. This isolate lacked 885 orthologous genes that were present in the rest of the strains (data not shown).

### 3.2 Sequence types and sample origin

A total of 64 *L. monocytogenes* isolates were analyzed (Supplementary Table S4). They were obtained from various types of cheeses, including soft cheese (*n* = 31), kneaded cheese (*n* = 7), chopped cheese (*n* = 3), unsalted cheese (*n* = 2), and curd (*n* = 1), but also from listeriosis cases, including blood (*n* = 19), and cerebrospinal fluid (*n* = 1) samples. These samples were collected from 2015 until 2018 and 8 Sequence Types (STs) were detected. Table 1 shows the STs among food and clinical samples. The predominant ST was ST2 (84.62% 55/65), and other STs were found in less than 4% of the samples. ST2 was the only ST found in food and clinical samples.

**Table 1:** *Listeria monocytogenes* sequence types (STs) among sample origin.

Sample origin	ST	*n (%)*
Food (cheeses)	ST2	42 (93.33)
	ST6	1 (2.22)
	ST796	2 (4.44)
Clinical	ST2	13 (65.00)
	ST1	1 (5.00)
	ST3	2 (10.00)
	ST4	1 (5.00)
	ST378	2 (10.00)
	ST392	1 (5.00)

### 3.3 Antimicrobial resistance

All the isolates were previously tested and showed susceptibility to penicillin, ampicillin, erythromycin, trimethoprim/sulfamethoxazole, and meropenem (Espinosa-Mata et al., 2021). Genetic determinants for AMR were also detected with WGS data from three databases, as shown in Table 2. The determinants *fosX* and *mprF_2* were found only by the CARD database, while GYRA_23 and TUFAB_7 were only found by Megares and *norB* and *tetS* were detected by 2 and 3 databases, respectively.

**Table 2:** Results of *Listeria monocytogenes* genetic determinants for antimicrobial resistance search from three public databases.

Determinant	CARD	Resfinder	Megares	*n*
FosX (fosfomycin)	1	0	0	65
GYRA_23^1^	0	0	1	63
MprF_2^2^	1	0	0	62
norB^3^	1	0	1	63
tetS (tetracycline)	1	1	1	4
tufAB_7^4^	0	0	1	65

^1^GYRA is a component of the bacterial DNA gyrase. Mutations in this protein may cause fluoroquinolones resistance (Lampidis, 2002).^2^MprF confers resistance to cell membrane disrupting cationic peptides (defensins) (Thedieck et al., 2006).^3^NorB confers resistance to fluoroquinolones and tetracycline (Alcock et al., 2023).^4^Mutations in tufAB (components of EF-Tu) may cause resistance to elfamycins (Prezioso et al., 2017).

Two isolates (Lm03 and Lmo22) presented 9 and 3 additional AMR determinants respectively, apart from the ones detected in most samples, and they lacked the *tetS* gene (Supplementary Table S4).

### 3.4 Genomic analysis

**Figure 1:**
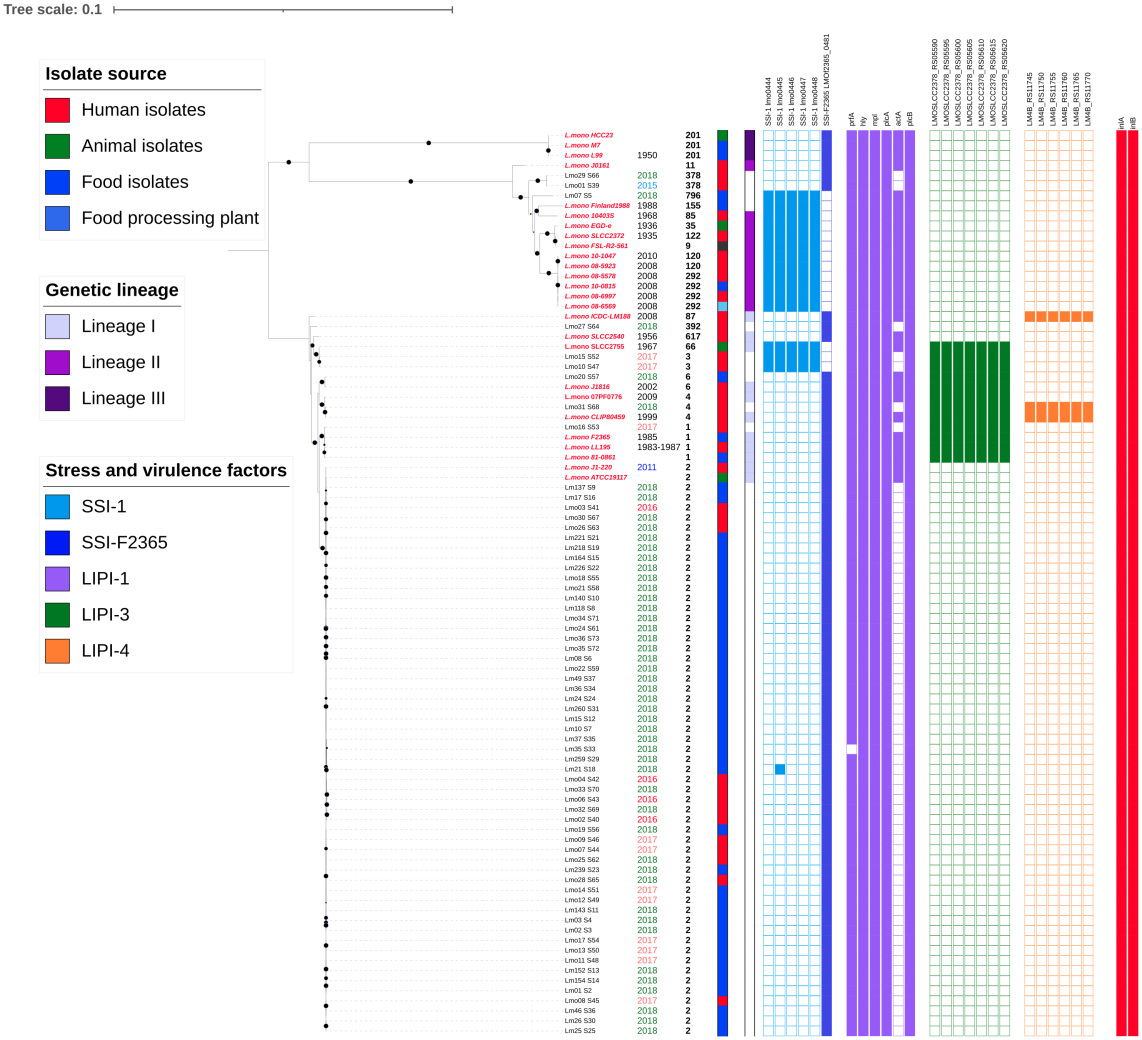
Phylogenetic tree from core genome alignment of 64 *L. monocytogenes* isolates and 26 references (2,112 genes are included). Reference sequence names are labeled in red and in italics. The isolation date and ST are indicated next to the isolates names. The isolate source, genetic lineage, and presence of SSI-1, SSI-F2365, LIPI-1, LIPI-3, and LIPI-4 are represented as binary data. Colored squares indicate presence. A digital version of the phylogenetic tree is available on iTOL: https://itol.embl.de/shared/epimol.

From the raw data, no plasmids or virulence determinants were found by PlasmidFinder and VirulenceFinder, respectively (data not shown). The core genome of all *L. monocytogenes* (*n* = 64) and references (*n* = 26) encompasses 2,112 genes spanning 1,896,388 nt with 175,308 informative sites (9.3% of complete sequence alignment). This alignment generated a maximum-likelihood tree shown in Figure 1, where bootstrapping values of 90 or higher are displayed with a black circle. Isolates were grouped according to the evolutionary lineages previously determined (Orsi et al., 2011). Most Ecuadorian samples were clustered in lineage I. The collection date, sequence type, isolate source, genetic lineage, presence of SSI-1, SSI-F2365, LIPI-1, LIPI-3, LIPI-4, and internalins were also depicted.

With respect to the stress survival islets, samples presented either SSI-1 or SSI-F2365, except for isolate Lm21, which presented SSI-F2365 but also one gene from SSI-1. Only 3 of our isolates (2 ST3 clinical isolates and 1 ST796 food isolate) contained SSI-1. None of the samples presented SSI-2, which is common in *L. innocua*.

Regarding the presence of pathogenicity islands and virulence factors, the isolates exhibited heterogeneous patterns. They all exhibited at least 5 genes from LIPI-1 (*prfA, plcA, hly, mpl, actA, plcB*), but all the references showed the complete island. Only two Ecuadorian isolates (Lm07 and Lmo20) harbored all genes from LIPI-1. Most of our isolates did not reveal the presence of gene *actA* or the percentage identities were lower than 75% with respect to the reference gene. Genes from LIPI-2, which are usually found in *L. ivanovii*, were absent from all the isolates. LIPI-3 was only present in 13 isolates from lineage 1 (7 references of ST66, ST6, ST4, and ST1). Clinical isolates Lmo15 (ST3), Lmo10 (ST3), Lmo31 (ST4), Lmo16 (ST1) and food isolate Lmo20 (ST6) presented all the genes from LIPI-3. LIPI-4 was present in only 3 isolates (2 references with ST87 and ST4 and Lmo31). Internalins A and B were present in all the isolates and references. All the isolates and references also showed the presence of genes associated with biofilm formation (*flaA, luxS, cheY*) (data not shown).

**Figure 2:**
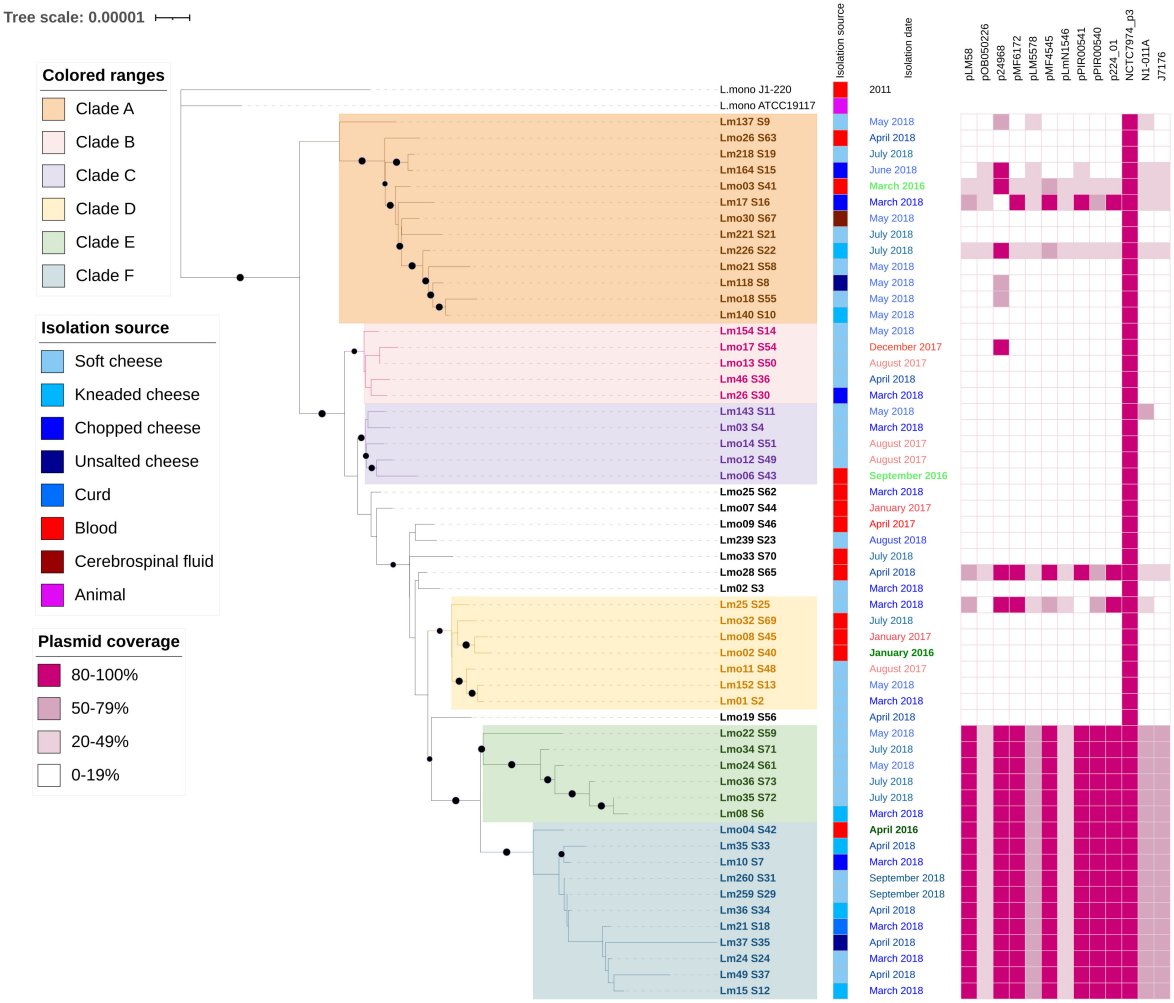
Phylogenetic tree from core genome alignment of 55 *L. monocytogenes* isolates and 2 references (2,112 genes are included) that belong to ST2. The isolation source, date, and place are indicated next to the isolate names. The plasmid coverage is presented in ranges (20–49%, 50–79%, and 80–100%). Colored squares indicate presence. P24968 corresponds to pl2015TE24968. P224_01 corresponds to pAUSMDU00000224. The digital version of the phylogenetic tree is available on iTOL: https://itol.embl.de/shared/epimol.

Since most of our samples belong to ST2, we obtained a more detailed phylogenetic tree based on the alignment of 57 ST2 strains, including two references, which is presented in Figure 2 (Bootstrap values ≥90% are shown in branches with a black circle). The multiple alignments included 2,376,722 bp with only 1,022 informative sites (less than 0.05% of the complete alignment). The isolation source and collection date are specified as metadata. Clades (A-F) are colored for better display. Strains in clade B and E were isolated only from cheeses, while the remaining clades included human and food samples. The presence of 13 plasmids is shown in ranges (20–49%, 50–79%, and 80–100% coverage) representing global similarity. All the isolates presented the whole NCRC7974_plasmid3, while clade E, part of F and Lm08_S6 (from clade B) showed the same plasmid pattern, an 80–100% coverage of plasmids pLM58, pl2015TE24968 (p24968), pMF6172, pMF4545, pPIR00541, pPIR00540, PAUSMDU00000224_01 (p224_01); 50–79% coverage of plasmids pLM5578, N1-011A and J1776; and 20–49% coverage of plasmids pOB050226 and pLmN1546.

**Figure 3:**
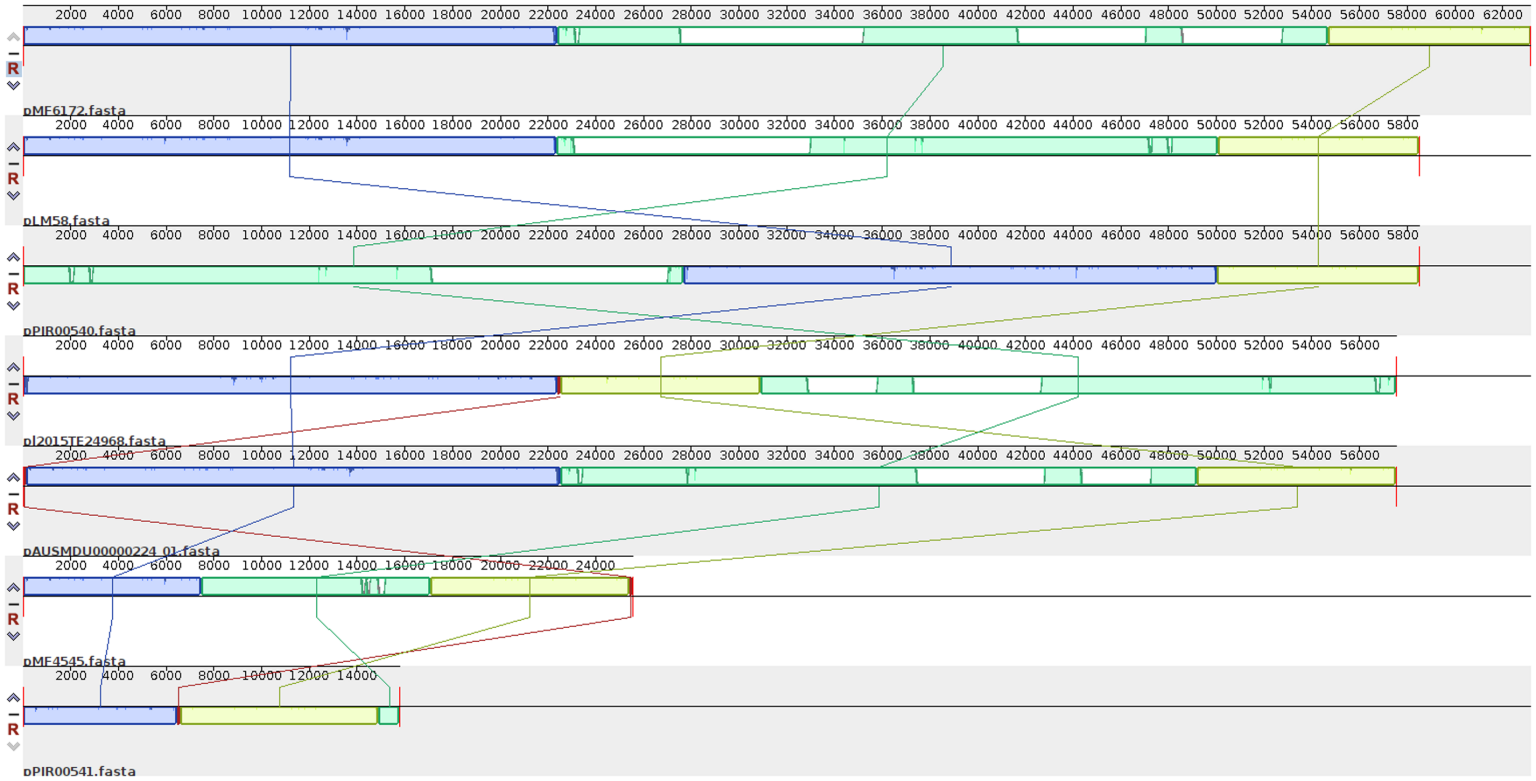
Locally collinear blocks (LCB) among the plasmids detected in Ecuadorian *L. monocytogenes* isolates. LBCs are colored according to the conserved fragments that are possibly homologous and free of internal genome rearrangements. LCBs with inverse orientation appear in the bottom of the center line.

When analyzing with more detail the pattern of plasmids present, we realized that many genes were present in most plasmids. Eleven genes, spanning 10 kb, were present in these 7 plasmids, while seven genes (8 kb) were shared by 6 plasmids, and 20 genes (14.7 kb) were present in 5 plasmids. Among these genes, we found six transposases, two DNA invertases, one DNA polymerase IV, one transposon resolvase, one NADH peroxidase, one protease, one gene involved in the regulation of Cadmium resistance, one Cadmium-transporting ATPase, and 24 hypothetical proteins (Supplementary Table S5). After the ANI calculation among the seven plasmids, we detected a high percentage of similarity (99.51–100%) in different sizes of fragments shared (9,743–50,589 bp) by every pair of plasmids (Supplementary Table S6). The alignment of the seven plasmids showed large LCBs that have lost synteny along the genomes, as seen in Figure 3. Two plasmids seem to have undergone inversions.

## 4 Discussion

This study provides important epidemiological information on *Listeria monocytogenes* in Ecuador. We have obtained complete genome sequences of *L. monocytogenes* isolates from various types of cheese from different provinces and human clinical cases. Of the total analyzed strains, 93.33% of cheese isolates and 65% of clinical isolates were ST2 (from clonal complex 2, CC2). This sequence type seems to be an important etiological agent of listeriosis in this country because, according to our data, more than half of the clinical cases from 2015 to 2018 from the National Institute of Public Health of Ecuador were caused by ST2 isolates. Further, other Ecuadorian *L. monocytogenes* isolated from illegally imported foods into the European Union were also identified as ST2 (3/3 isolates from dairy and meat products) (Rychli et al., 2018). ST2 strains have been also isolated from dairy, meat, and fish products in Albania, Argentina, Georgia, the Republic of Moldova, Peru, and Ukraine (Rychli et al., 2018), from ready-to-eat fish, meat, sausage, and chicken products in other European countries (Nielsen et al., 2017), from meat products in South Africa (Mafuna et al., 2021), and from food and clinical cases in Chile (Toledo et al., 2018), in USA, and from food in China (Yin et al., 2015). Isolates from CC2 have been previously associated with listeriosis cases with few or no immunosuppressive comorbidities (Maury et al., 2016), and with sporadic but no outbreak-related cases (Nielsen et al., 2017). CC2 strains have also been isolated from a cheese plant as the second most prevalent CC, and the isolates obtained from the plant environment grouped in different clusters than the cheese isolates (Nielsen et al., 2017), suggesting a high genetic diversity among environmental isolates.

Apart from ST2, other STs were recovered from food samples: ST6 (lineage I, soft cheese, 2008) and ST796 (lineage II, kneaded cheese, 2018). An ST6 outbreak strain caused 1,060 listeriosis cases in 2017-2018 in South Africa, with a case-fatality rate of 27%. The implicated strain was found in polony samples, a ready-to-eat processed meat product, but also in environmental swabs of the producing plant (Smith et al., 2019). In Europe, another ST6 strain was implicated in an outbreak with 21 cases (3 deaths and 1 miscarriage) in the Netherlands and Belgium during 2017-2019 for the consumption of ready-to-eat meat products (ECDC and EFSA, 2019). In Chile, an ST6 strain was isolated from the environment in a food plant (Toledo et al., 2018), and, in France, an ST6 was recovered from turkey deli meat (Yin et al., 2015). On the other hand, there are no records on ST796 strains at https://bigsdb.pasteur.fr/listeria/.

Strains from ST1, ST3, ST4, ST378, and ST392 were recovered only from clinical cases. ST1 strains have also been isolated from meat products in South Africa (Mafuna et al., 2021), from food and clinical samples from Chile (Toledo et al., 2018), from food causing outbreaks in China, USA, and Austria (Yin et al., 2015). ST3 strains have been recovered from food in Chile (Toledo et al., 2018), in France and China, and clinical samples in China (Yin et al., 2015). Strains from food in Italy, sporadic cases in the USA, food in France (Yin et al., 2015), and also from clinical cases in Ireland (Hilliard et al., 2018) have been identified as ST4. ST392 isolates have been obtained from clinical cases in Chile (Toledo et al., 2018). This information, along with the fact that we only analyzed cheese samples, suggests that the possible source of infection in clinical cases of these STs may be other food products.

The AMR analyses showed phenotypic susceptibility patterns to all antibiotics tested in all the Ecuadorian isolates. With the bioinformatic prediction of AMR, we found some determinants in different databases. With CARD, we found the presence of *fosX*, which is explained because *L. monocytogenes* are naturally resistant to fosfomycin (Luque-Sastre et al., 2018), and *mprF*. This latter determinant allows the bacteria to resist CAMPs (cationic antimicrobial peptides), components of the host immune response (Thedieck et al., 2006). When using Megares as a reference database, we detected the presence of Gyra_23 and TUFAB-7, which may confer resistance to fluoroquinolones and efamycins, respectively. However, in a previous study, mutations on *gyrA* were obtained with no effect on the transformed *L. monocytogenes* strains in their resistance to nalidixic acid (Lampidis, 2002). While working on this article, we did not find any previous publications on *Listeria* and efamycin resistance. We found *norB* and *tetS* in several databases. *L. monocytogenes* strains harboring *norB, fosX, lin, tetS,* and *mprF* have been identified in South Africa (Mafuna et al., 2021).

The number of antimicrobial resistance determinants identified differs among the 3 considered databases. CARD is based on BLAST (Basic Local Alignment Search Tool) against its own database. ResFinder is based on BLAST and KMA (k-mer alignment) against its own database. MEGARes is based on BWA (Burrows-Wheeler Aligner) against a database derived from ARG-ANNOT, CARD, NCBI, Lahey Clinic beta-lactamase archive, and ResFinder (Hendriksen et al., 2019). In a study comparing CARD, ARG-ANNOT, and Resfinder with different antimicrobial resistance identifiers, the largest number of genes was obtained when using CARD as the reference database (Doyle et al., 2020). Differences in the outcome of AMR prediction may be due to the default settings for percentage of identity and gene length on the BLAST search of each program, or the curation of the databases (Hendriksen et al., 2019). With the differences obtained depending on the chosen database, there exists a need to standardize the pipelines and the databases.

Among stress factors, we found SSI-1 in 3 isolates, Lm07 (ST796) from lineage II, isolated from food, Lmo10 (ST3), and Lmo15 (ST3) from lineage I, isolated from human cases. This five-gene islet was also present in 11 references from lineage II, isolated from human, animal, food, and food processing plant samples, and in one reference from lineage I, isolated from an animal sample. These isolates belong to ST9, 35, 85, 66, 120, 122, 155, and 292. Deletion of the islet has shown decreased growth at suboptimal conditions, such as high salt concentrations and low pH (Ryan et al., 2010; Hein et al., 2011), and bile and gastric stresses (Harter et al., 2017). SSI-1 has also been associated with robustness and adherence of biofilm at 30°C (Keeney et al., 2018). The majority of our isolates lacked SSI-1 but instead contained SSI-F2365, an ORF that is transcribed in the opposite direction in the same location (Ryan et al., 2010), with an unknown function (Harter et al., 2017).

We also looked for SSI-2, two homologs of *lin0464* and *lin0465* present in *L. innocua* that are also transcribed in opposite directions (Harter et al., 2017). None of our *L. monocytogenes* isolates contained SSI-2. Since these two genes are usually present in ST121 strains (Hein et al., 2011) that persist well in food processing plants for months and even years, it is suggested that the presence of SSI-2 in *L. monocytogenes* provides tolerance to alkaline and oxidative stress (Harter et al., 2017), different to the ones provided by SSI-1. An earlier investigation reported the prevalence of these 3 inserts. SSI-1 (33.3%), SSI-2 (11.8%), and SSI-F2365 (54.8%) were evaluated in 476 *L. monocytogenes* isolates from foods, food-processing environments, and humans from 37 countries from America, Europe, Asia, and Africa (Harter et al., 2017).

We also evaluated these 3 insertions in non-*monocytogenes Listeria* isolates. SSI-1 was found in *L. welshimeri* and SSI-2 was present in all five *L. innocua* isolates. All *L. innocua, L. seeligeri, L. welshimeri* isolates lacked SSI-F2365, but it was present in most of our *L. monocytogenes* isolates, suggesting importance to this pathogenic species.

The complete LIPI-1 island was detected in all *L. monocytogenes* references. These six physically linked genes are responsible for crucial steps in the intracellular life cycle of this species (Vázquez-Boland et al., 2001). Nevertheless, the *actA* gene was absent (or it presented premature stop codons) in all Ecuadorian ST2, ST3, ST4, and ST392 isolates, but is present in the references from the same STs. The *actA* gene is responsible for actin polymerization and motility within the cytoplasm of the host cell, for biofilm formation (Travier et al., 2013), and for cell-to-cell spread (Vázquez-Boland et al., 2001). This gene was also missing from ST2 strains isolated from the food chain in South Africa (Mafuna et al., 2021). Another study that characterized 100 *L. monocytogenes* isolates from environment and food samples in 3 food plants over a 4-year period found that 33% of the isolates contained a deletion or truncation of *actA*, suggesting that these isolates may present reduced intracellular mobility (Hurley et al., 2019).

The complete LIPI-3 was found in 5 Ecuadorian isolates (from food and clinical samples) and 7 references (animal, food, and clinical) with no monophyletic clustering which suggests independent gains in different isolates. LIPI-3 includes an inducible haemolytic and cytotoxic peptide and virulence enhancer, which is known as listeriolysin S (Cotter et al., 2008). Strains harboring LIPI-3 belong to ST1, 3, 4, 6, and 66, from lineage I. In South Africa, 21.7% of isolates from meat products (from lineage I and II) harbored LIPI-3 (Matle et al., 2020).

The only isolate containing LIPI-4 was an ST4 strain isolated from blood from a 4-day-old baby. This pathogenicity island in ST4 strains is recognized as the first virulence factor specifically associated with the central nervous system and maternal-neonatal listeriosis since the presence of LIPI-4 enhances bacterial tropism to placentas and fetuses of pregnant humanized mice, and also contributes to neuroinvasion (Maury et al., 2016). LIPI-4 has been usually associated with ST4 and CC4 strains (Hilliard et al., 2018). Nevertheless, strains from CC2 and CC87 presented this island in China and South Africa (Matle et al., 2020), and ST1 and ST204 strains also harbored LIPI-4 in South Africa (Mafuna et al., 2021).

In all Ecuadorian *L. monocytogenes* isolates, complete *inlA* and *inlB* genes were detected. Some studies have shown that the presence of premature stop codons in *inlA* is associated with reduced ability to invade human intestinal epithelial cells *in vitro* (Su et al., 2019), like in 31% of *L. monocytogenes* isolates from food processing-plants in Ireland (Hurley et al., 2019).

All our isolates harbored *flaA, luxS,* and *cheY* genes. *flaA* gene, coding for flagellin A, has been identified as important for biofilm formation since motility may be required for surface attachment to biotic and abiotic surfaces (Warke et al., 2017). The gene *luxS* has been also associated with biofilm formation because it encodes S-ribosylhomocysteine. This enzyme is a precursor of autoinducer-2 in pathogenic bacteria and *luxS* mutants form biofilms with changed architecture or do not form any at all (Vendeville et al., 2005). *cheY* is part of a chemotactic system with two functions: it regulates the response to external signals, and it is also responsible for the flagellum rotor regulation. A *cheY* mutant presented a loss of motility activity and the biofilm formation was lower than the one formed by the wild type strain (Fan et al., 2020). The fact that our results showed the presence of these 3 genes in all Ecuadorian isolates suggests a high capacity of these *L. monocytogenes* strains to form biofilm, which may be important for cross-contamination after thermal treatment applied in foods or during storage at refrigeration or freezing temperatures.

Among the ST2 isolates, we determined sublineages with wide distribution in time (at least from two different years of isolation) and types of samples (food and clinical isolates). Clades B and E included only cheese isolates, while clades A, C, D, and F grouped isolates from food and clinical samples.

Isolates from clade A showed the greatest diversity in sample origin (blood and cerebrospinal fluid for clinical samples and 4 different types of cheeses). All isolates were collected in 2018, except for a clinical case from 2016. Clade F showed the greatest nucleotide divergence along the core genome of 11 isolates, as seen in Figure 2. When comparing these genomes, 565 variable sites were found.

Clade E and F include isolates harboring the same plasmid pattern, suggesting a plasmid gain in their common ancestor and a subsequent plasmid loss in the 4 clinical isolates at the bottom of the tree (Lmo33, Lmo06, Lmo32, and Lmo02). Food isolates from these clades were collected only in 2018, while clinical isolates in 2016 and 2018. There is only one isolate from another clade (B) that presented the same plasmid pattern, Lm08, collected in 2018, suggesting an independent horizontal gene transfer event.

In South Africa, ST2 strains with N1-011A and J1776 have been identified, and the presence of pLM5578 was found only in ST121 and ST321 isolates (Mafuna et al., 2021). We did not detect ST121 and ST321 strains.

Although it might seem that these strains contain several plasmids, the results of the comparative analysis suggest that the plasmids share the same backbone and have great plasticity of gene gain and loss.

Almost the complete NCTC7974_plasmid3 was present in all Ecuadorian isolates (more than 91% of the plasmid length). The length is 593,685 nucleotides with 568 CDSs, from which 164 are hypothetical proteins. There is no published information about this plasmid. We also found the presence of this plasmid, with different coverage lengths, in non-*monocytogenes* isolates: 66.58% in the only *L. seeligeri* strain, from 82.98 to 95.07% in 5 *L. innocua* strains and 95.09% in the only *L. welshimeri* isolate (data not shown). After this plasmid annotation with Prokka (Seemann, 2014), we found the presence of housekeeping and accessory genes (Supplementary Table S7). Some noteworthy genes to mention are tRNAs, 5S, 16S, and 23S ribosomal RNA, 30S and 50S ribosomal proteins, ATP synthase subunits, DNA gyrase, DNA topoisomerase, cell division proteins, chromosomal replication initiator protein and partition protein, phosphoglycerate kinase, CRISPR with 32 repeat units and Cas1, Cas2, Cas9, and Csn2, but also internalins J. The latter has been associated with virulence in *L. monocytogenes* (Sabet et al., 2005). In *Rhizobium* species, a plasmid with housekeeping genes important for pantothenate synthesis has been identified. The authors suggested an intragenomic transfer from the rhizobial chromosome to the plasmid (Villaseñor et al., 2011), and this might also be the case for this plasmid and *L. monocytogenes*.

The high genetic similarity between ST2 isolates from cheeses and clinical cases suggests the spread of *L. monocytogenes* through the food chain (in this case, cheese consumption) to humans in Ecuador and the need for surveillance of the listeriosis in this country. We also found a few isolates with virulence factors and stress survival islets, which in general means that Ecuadorian isolates show lower pathogenic potential, but a wider and bigger analysis with other types of foods is required.

In Ecuador, listeriosis is not a notifiable disease and there is not a National Surveillance Program for this foodborne pathogen. Although this study provides valuable genomic and epidemiological information on *L. monocytogenes*, further investigations on other contamination sources apart from cheeses and plant production facilities are still necessary. Genotyping of food, plants, and clinical isolates is recommended for routine surveillance to infer epidemiological links and the adoption of control measures to prevent listeriosis.

## Data availability statement

The datasets presented in this study can be found in online repositories. The names of the repository/repositories and accession number (s) can be found in the article/Supplementary material.

## Ethics statement

Ethical approval was not required for the studies involving humans because we had access to bacterial strains from clinical samples obtained by the National Institute of Public Health Research in Ecuador. The studies were conducted in accordance with the local legislation and institutional requirements.

## Author contributions

LM: Conceptualization, Formal analysis, Investigation, Methodology, Writing – original draft. EE-M: Investigation, Writing – review & editing. AF: Investigation, Writing – review & editing. SZ: Conceptualization, Writing – review & editing. FG-C: Conceptualization, Funding acquisition, Writing – review & editing.
